# Superlubricity of Graphite Induced by Multiple Transferred Graphene Nanoflakes

**DOI:** 10.1002/advs.201700616

**Published:** 2018-01-03

**Authors:** Jinjin Li, Tianyang Gao, Jianbin Luo

**Affiliations:** ^1^ State Key Laboratory of Tribology Tsinghua University Beijing 100084 China

**Keywords:** graphene, graphite, nanotribology, superlubricity, transferred nanoflakes

## Abstract

2D or 3D layered materials, such as graphene, graphite, and molybdenum disulfide, usually exhibit superlubricity properties when sliding occurs between the incommensurate interface lattices. This study reports the superlubricity between graphite and silica under ambient conditions, induced by the formation of multiple transferred graphene nanoflakes on the asperities of silica surfaces after the initial frictional sliding. The friction coefficient can be reduced to as low as 0.0003 with excellent robustness and is independent of the surface roughness, sliding velocities, and rotation angles. The superlubricity mechanism can be attributed to the extremely weak interaction and easy sliding between the transferred graphene nanoflakes and graphite in their incommensurate contact. This finding has important implications for developing approaches to achieve superlubricity of layered materials at the nanoscale by tribointeractions.

## 1 Introduction

Friction and wear are the two major sources of considerable energy consumptions in mechanical sliding systems, especially in the nano and micromachines.^1^ The superlubricity, a physical regime in which the friction between two sliding surfaces nearly vanishes (or the sliding friction coefficient is in the level of 0.001),^2^ is one of the most effective approaches to reduce the frictional energy dissipation and meanwhile provide a near‐wearless condition. There are a series of solid lubricants, such as the diamond‐like carbon (DLC) film and 2D or 3D layered materials, including graphene, graphite, boron nitride, and molybdenum disulfide (MoS_2_), that have been observed to achieve the superlubricity state under certain special conditions.^3^ For example, Martin et al. observed the superlow friction of a MoS_2_ coating in the ultrahigh vacuum, where the atomic origin of superlubricity was from the incommensurate contact of MoS_2_ basal planes.^4^ Dienwiebel et al. studied the friction between the graphite nanoflake and sheet, observing the rotation angle dependent superlubricity phenomenon.[[qv: 3c,5]] Liu et al. found that the micrometer‐scale graphite flakes were retracted back to their initial positions after displacement from the equilibrium configuration due to the structural superlubricity between graphite layers in the sliding direction.[[qv: 3d,6]] Feng et al. observed the facile translational and rotational motions between graphene nanoflakes (GNFs) and graphene surface at an extremely low temperature.^7^ Only recently, superlubricity has also been achieved for dissimilar surfaces, like amorphous antimony or crystalline gold nanoparticles sliding on graphite,^8^ graphene nanoribbons sliding on gold,^9^ and in tribometer sliding experiments between DLC and graphene by the formation of graphene nanoscrolls surrounding the nanodiamond particles.^10^

Among these superlubricity systems, one of the most impressive materials is graphite, which has been widely used as a solid lubricant in mechanical devices. Its lamellar structure and very weak van der Waals (vdW) interactions between atomic layers facilitate the sliding between adjacent layers, which is the origin of the superlubricity characteristics.[[qv: 3d,5]] However, there exists a limitation that the superlubricity of graphite can only be achieved with some specific materials, such as layered materials (graphite or graphene) or inert metal (like gold) nanoparticles under very low load.[[qv: 3d,8b]] The friction force usually increases several times and even more if the sliding occurs between graphite and common materials (nonlayered).[[qv: 2b,3f]] To solve this limitation, a novel method was proposed to achieve the robust superlubricity between graphite and nonlayered material silica (silica was chose here because its probe is easily fabricated and widely used in nanotribology); that is, forming the multiple transferred GNFs on the asperities of the silica surface through the frictional sliding (presliding) on the graphite. This method provides the direct experimental evidence for the superlubricity between graphite and nonlayered materials via the tribointeractions. Therefore, in this study, the superlubricity behavior between graphite and silica achieved by this method was studied and its efficient lubrication mechanism was revealed at the nanoscale.

## 2 Results and Discussion

**Figure 1:**
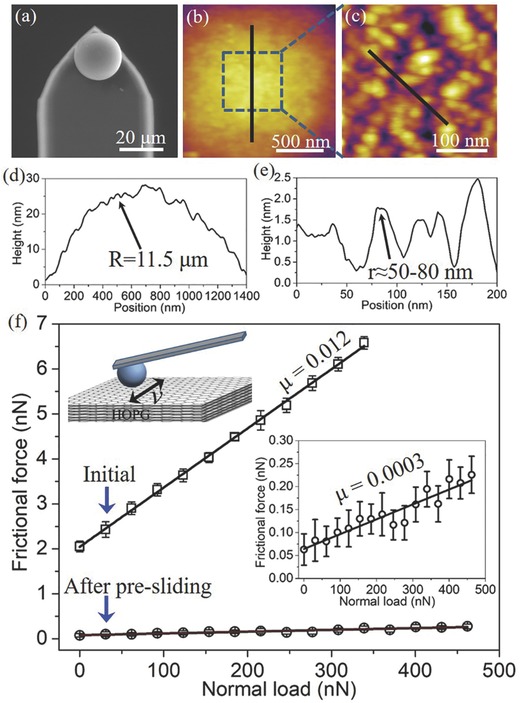
a) Scanning electron microscopy image of the silica particle attached to the AFM cantilever end. b) AFM image of the topography on the top central region of the SiO_2_ probe. c) Enlarged view of the topography in the dashed square marked in (b). d) Cross‐sectional height profile of the probe surface on the position of the black line in (b). e) Cross‐sectional height profile of the probe surface on the position of the black line in (c). f) Frictional forces as functions of normal loads between the probe and HOPG, measured before and after the presliding, respectively. The sliding speed was set as 2.4 µm s^−1^. The full lines are the linear fits to these points, giving the friction coefficients of 0.012 (initial) and 0.0003 (after the presliding). The inset is the illustration of the probe sliding on HOPG during the frictional force measurement and the enlarged view of the frictional force versus normal loads after the presliding.

The silica particle with a radius of 11.5 µm was first glued onto a rectangular tipless cantilever end by using epoxy glue, yielding a SiO_2_ probe (**Figure** **1**a). The topography on the top region of the probe was obtained by the atomic force microscopy (AFM), as shown in Figure 1b,c. The surface of the probe was not atomically smooth, and instead a series of asperities appeared on the surface with a roughness of ≈1 nm over an area of 90 000 nm^2^. From the cross‐sectional height profile in Figure 1d,e, the radii (*r*) of these asperities were in the range of 50–80 nm, with a peak‐to‐peak distance of ≈30 nm. Meanwhile, a highly ordered pyrolytic graphite (HOPG, 0.4° mosaic spread) substrate was freshly cleaved by using the adhesive tape to give a clean atomically flat surface. The frictional forces as functions of normal loads were measured on AFM, by driving the probe sliding on the freshly cleaved HOPG as the load was increased gradually, as shown in Figure 1f.

At the initial stage of measurement, the frictional forces maintained high values and exhibited a linear relation with the applied load. The friction coefficient (μ) was approximately μ = 0.012 ± 0.003, obtained from the slope of the linear fitting line to these points. It was observed that there was an offset frictional force when the applied load was zero, indicating that there exists a strong adhesive force between the probe and HOPG. Thereafter, the probe was compelled to slide on the HOPG with a scanning area of 10 × 10 µm^2^ at a load of 185 nN and a velocity of 20 µm s^−1^ continuously. After this initial sliding (presliding; see the Experimental Section), the frictional force reduced suddenly to close to zero (Figure 1f), and it remained this superlow value when the scanning area was varied from 0.6 × 0.6 to 20 × 20 µm^2^. It should be mentioned that when the probe slid across the atomic steps on graphite layers (due to the different thickness of cleaved layer), there would appear friction peaks caused by the geometric effect,^11^ but it did not have influence on the superlow friction when the probe slid on the atomically smooth area (Figure S1, Supporting Information). Figure 1f (inset) shows that the superlow frictional forces also exhibited a linear relation with applied load. The friction coefficient was approximately μ = 0.0003 ± 0.0001, obtained from the slope of the linear fitting line, which entered the superlubricity regime (μ ≈ 0.001).[[qv: 2b]] In addition, the offset frictional force at zero load was close to 0, indicative of the very small adhesive force between two friction surfaces after the presliding.

**Figure 2:**
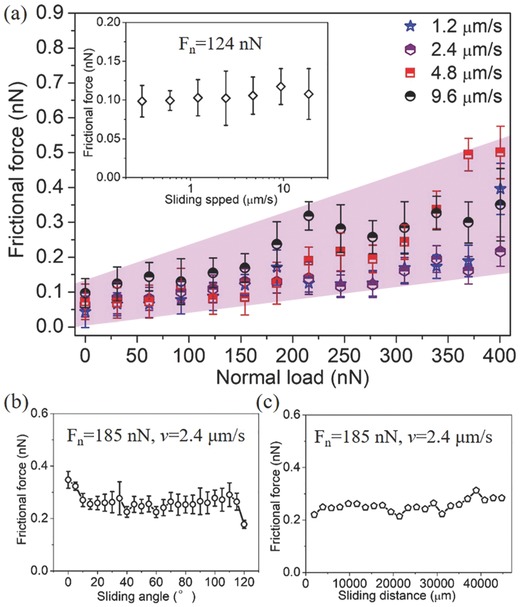
a) Frictional force as a function of normal load between the probe and HOPG under four different sliding speeds (1.2, 2.4, 4.8, and 9.6 µm s^−1^). The inset is the relationship between the frictional force and sliding speed at a constant normal load of 124 nN. b) Relationship between frictional force and sliding angle (varied from 0° to 120°) at a constant normal load of 185 nN and a sliding velocity of 2.4 µm s^−1^. c) Relationship between frictional force and sliding distance (measured continuously) at a constant normal load of 185 nN and a velocity of 2.4 µm s^−1^.

Because the superlubricity of graphite can be greatly influenced by the sliding velocity, distance, and rotation angle, the relationship between the superlubricity behavior and these parameters was investigated, as shown in **Figure** **2**. The frictional forces versus loads were first measured under four different scanning speeds, respectively (Figure 2a), with the results showing that the frictional behaviors under these different scanning speeds were almost the same. All the frictional forces maintained superlow values with small fluctuations until the load increased to 400 nN, giving friction coefficients of 0.0003–0.0007. The relationship between the frictional force and sliding velocity at a constant normal load of 124 nN is shown in the inset of Figure 2a. The frictional forces always fluctuated within the range of 0.09–0.13 nN with the sliding velocity varying from 0.3 to 18.7 µm s^−1^, confirming that the frictional force in the superlubricity regime is independent of the sliding velocity.

The scanning angle of the probe was varied from 0° to 120° to give different sliding orientations on the HOPG, and the frictional force was measured at an interval of 5°at a constant normal load of 185 nN (Figure 2b). It was observed that the frictional forces remained superlow values (fluctuated within the range of 0.2–0.35 nN) at each sliding orientation. It has been demonstrated that the superlubricity of graphite sheets would disappear suddenly at two certain sliding orientations with an angle difference of 60°, because the commensurate contact (leading to high friction) is consistent with the periodicity of the graphite lattice.[[qv: 3c,d]] However, we did not observe the angle‐dependent superlubricity behavior of graphite here, indicative of the difference from the superlubricity system of graphite sheets. Meanwhile, the frictional force was also measured at the constant normal load of 185 nN continuously with a long sliding distance of up to 45 000 µm, as shown in Figure 2c. There was no great increase in the frictional force with increasing the sliding distance, and the ratio of frictional forces to normal load (equal to μ approximately) was always in the level of 0.001, exhibiting the excellent robustness of the superlubricity state achieved by the presliding on HOPG.

As there are only two kinds of materials (graphite and silica) in the superlubricity system, the interactions between graphite and silica during the presliding process play the key role in friction reduction. It is impossible to produce new materials by the tribochemical reaction between graphite and silica because this reaction requires very high temperature.^12^ Many previous studies found that the outermost layers on the graphite, graphene, or MoS_2_ can be transferred onto the other materials, such as tungsten, steel, and ceramics, by the tribointeractions between them.[[qv: 3a,c,13]] It is because the vdW interaction between interlayers of these layered materials is very weak, which facilitates the transfer process. Therefore, we inferred that parts of the outermost layers on the graphite were the most likely to be exfoliated and transferred onto the asperities (contact points) of the silica surface by the tribointeractions, which can create a beneficial condition for the significant reduction of frictional force.

**Figure 3:**
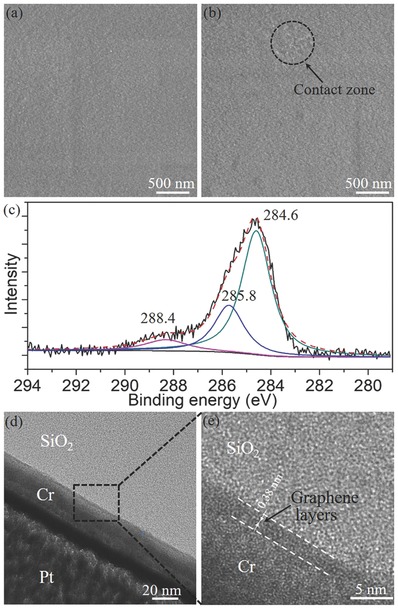
a) FESEM image of the topography on the top center of the probe before the presliding. b) FESEM image of topography on the top center of the probe after the presliding. c) XPS of C 1s spectra from the top center surface of the probe after the presliding, fitted with three Lorentzian–Gaussian peaks. d) HRTEM images of the cross‐section of the top center surface of the probe after the presliding. e) Enlarged view of the HRTEM image in the black dashed square marked in (d). Pt and Cr films are deposited as the protective film during TEM sample preparation.

To confirm this hypothesis, the topographies of the probe before and after the presliding were investigated by the field emission scanning electron microscope (FESEM), as shown in **Figure** **3**a,b. There were no obvious differences between the two topographies of the probe. Meanwhile, the topography of the probe was also studied by AFM after the presliding, showing a similar topography to that in Figure 1c (the roughness was close to 1 nm). These results indicate that there was no obvious damage and wear occurring on the probe surface after the presliding. However, The FESEM result shows there was something located on the probe dispersedly within a diameter of 500 nm after the presliding (Figure 3b), which is considered as the contact zone. The chemical composition on the probe after the presliding was detected by X‐ray photoelectron spectroscopy (XPS), as shown in Figure 3c. The C 1s spectra have three peaks at 284.6, 285.8, and 288.4 eV, fitted with three Lorentzian–Gaussian peaks. These peaks are attributed to the sp^2^ carbon bonds, sp^3^ carbon bonds, and O—C=O bonds, respectively, which is in accordance with the C 1s spectra of pristine graphene.^14^ It should be noted that the spectra from the adventitious carbon contamination coexisted with that of graphene. Therefore, the cross‐sectional high‐resolution transmission electron microscope (HRTEM) was also used to image the structure of the transferred nanoflakes located in the contact zone after the presliding, as shown in Figure 3d,e. It is found that there was a transfer film with a thickness of 2.3 nm covered on the probe surface. Some weak features existed in Figure 3e show that the film is a composite structure with multiple layers mixed with amorphous structures. It can be inferred that the transferred GNFs were only adsorbed on the contact asperities by the tribointeractions. The spacing (*d*_s_) of the multiple layers was about *d*_s_ = 0.38 nm, a little larger than that of pristine graphite (*d*_s_ = 0.33 nm),^15^ which may be attributed to the introduction of oxygen‐containing functional groups during the tribo‐exfoliated process.

**Figure 4:**
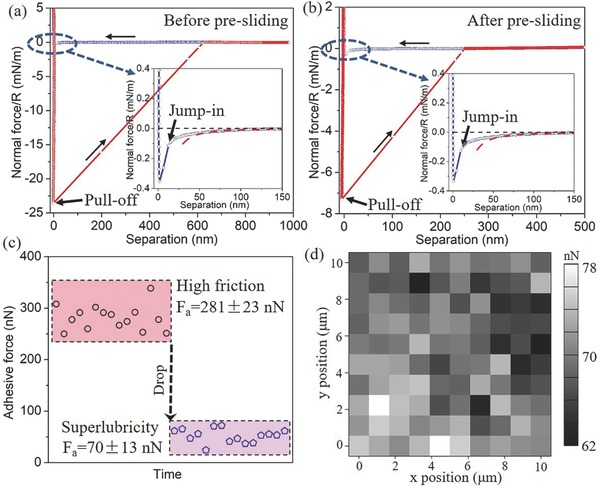
a) Normal force/*R* as a function of separation between the probe and HOPG (approach and retrace) before presliding. The inset is the enlarged view of the normal force as the probe approached HOPG, showing the jump‐in behavior. The red dashed line is the fitting line based on Equation (1). b) Normal force/*R* as a function of separation between the probe and HOPG (approach and retrace) after the presliding. The inset is the enlarged view of normal force as the probe approached HOPG. The red dashed line is the fitting line based on Equation (1). c) Adhesive forces in the high friction regime (before presliding) and superlubricity regime (after presliding) over 15 independent measurements (different probe and freshly cleaved HOPG). d) Adhesive force map in an area of 10 × 10 µm^2^ extracted from 100 normal force curves as the probe retracted from HOPG after the presliding.

The above frictional results and surface analysis of the probe indicate that it is highly likely to form the GNFs on the asperities of the silica surface after the presliding. In order to verify this further, the normal forces versus separation were measured before and after the presliding as the probe first approached HOPG and then detached from the HOPG, as shown in **Figure** **4**a,b. When the probe approached the HOPG, the normal force behaviors before and after the presliding were almost the same; that is, there appeared an obvious attractive force as the separation reduced to 150 nm, which originates from the vdW force between two friction surfaces, and can be expressed by Equation (1), 16

(1) $$F_{\mathrm{vdW}}=-\frac{AR}{6D^{2}}$$

where *R* is the radius of the probe, *A* is the Hamaker constant that depends on the dielectric properties of the two surfaces, and *D* is the separation between two surfaces. By fitting the vdW force with Equation (1), the ratio of the Hamaker constant after the presliding to that before presliding is about 1.18, indicating that the dielectric properties of the probe did not undergo a great change. When the probe detached from the HOPG, the two surfaces were jumped out of the contact until the pulling force exceeded the critical pull‐off value (adhesive force). However, there are great differences between the adhesive forces in the two cases. The adhesive force after the presliding is approximately one fourth of that before presliding. To eliminate the accidental factor from the probe, we carried out over 15 independent measurements (different probes and freshly cleaved HOPG) for the adhesive force before and after the presliding, as shown in Figure 4c. The data show that all the adhesive forces after entering the superlubricity regime by presliding (*F*_a_ = 70 ± 13 nN) were much lower than that in the high friction regime before presliding (*F*_a_ = 281 ± 23 nN), which indicates that the adhesive force between the probe and HOPG was significantly reduced during the presliding process. It is in accordance with the change in the offset frictional force (at zero load) in Figure 1f. Figure 4d shows the adhesive force map in an area of 10 × 10 µm^2^, extracted from 100 normal force curves after the presliding. It was seen that all the adhesive forces maintained low values and varied slightly (<12%) over the entire measured region, indicating that the adhesive force after entering the superlubricity regime is independent of the contact positions of HOPG.

According to the Johnson, Kendall, and Roberts theory, the adhesive force between two surfaces can be achieved by Equation (2), 16

(2) $$F_{a}=\frac{3\pi RW_{\mathrm{AB}}e^{-\sigma /\sigma_{0}}}{2}$$

where *R* is the radius of the probe, σ is the roughness of the probe, σ_0_ is the constant, and *W*_AB_ is the dispersive work of adhesion per unit area of surface A in contact with surface B (A: asperities on the probe; B: graphite), which can be expressed by Equation (3), 17

(3) $$W_{\mathrm{AB}}=2\sqrt{\gamma_{A}^{d}\gamma_{B}^{d}}$$

**Figure 5:**
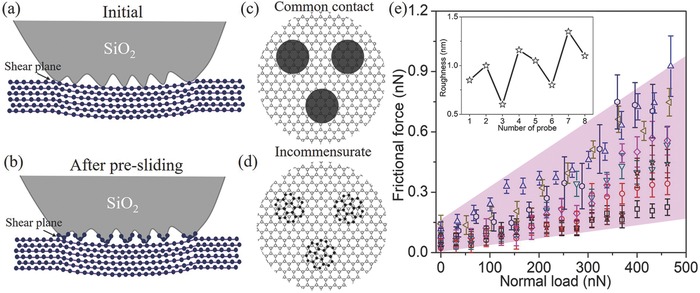
a) Illustration of the shear plane between the silica asperities and graphite at the initial stage. b) Illustration of the transferred GNFs on the asperities of the silica surface and the shear plane after the presliding. c) Illustration of the common contact between silica asperities and graphite at the initial stage. d) Illustration of the incommensurate contact between the transferred GNFs and graphite after the presliding. e) Frictional forces as functions of normal loads between eight different probes (random roughness) and HOPG after the presliding. The sliding speed was set as 2.4 µm s^−1^. The inset is the roughness of the eight different probes over an area of 90 000 nm^2^.

Where $\gamma_{A}^{d}$ and $\gamma_{B}^{d}$ are the surface energies of surface A and B, respectively. In the case of silica and graphite, their surface energies have been measured at ambient conditions by many researchers, which gives the surface energy of graphite and silica as $\gamma_{\mathrm{gra}}^{d}\approx 54.8\;\mathrm{mJ}\;m^{-2}$ and $\gamma_{{\mathrm{sio}}_{2}}^{d}\approx 600\;\mathrm{mJ}\;m^{-2}$.^18^ Thus, the interfacial energy between silica and graphite can be calculated as *W*_gra‐sio2_ ≈ 363 mJ m^−2^. As the radius and roughness of the probe remained constant during the sliding process, the reduction in *W*_AB_ is the only explanation for the significant reduction in the adhesive force after the presliding. In this case, the dispersive work of adhesion after the appearance of superlubricity (*W*_AB–sup_) can be obtained by the following equation

(4) $$W_{\mathrm{AB}-\sup}=\frac{F_{a-\sup}}{F_{a}}W_{\mathrm{AB}}$$

where *F*_a_ and *F*_a–sup_ are the measured adhesive forces before and after the presliding. Based on Equation (4), the value of *W*_AB–sup_ was calculated as *W*_AB–sup_ ≈ 91 mJ m^−2^. Combined with Equation (3), the surface energy of the asperities (in the contact zone) on the probe after the presliding was obtained as $\gamma_{{\mathrm{sio}}_{2}-\sup}^{d}\approx 38\;\mathrm{mJ}\;m^{-2}$, which becomes about one sixteenth of its original value. The value of $\gamma_{{\mathrm{sio}}_{2}-\sup}^{d}$ is consistent with the surface energy of graphene at ambient conditions,[[qv: 18a]] confirming that there are GNFs exfoliated from the outermost layer of graphite via the tribointeractions and transferred onto the asperities of the silica surface after the presliding (**Figure** **5**b).

According to these analyses, it is clear that the transfer of GNFs onto the asperities of the silica after the presliding plays the key role in the achievement of superlubricity. Many previous studies demonstrate that the interaction between two graphite sheets is extremely weak and they can easily slide with each other once they are in the incommensurate contact.[[qv: 3d,5]] Therefore, the realization of a stable incommensurate sliding contact is the precondition required to achieve superlubricity of graphite. At the initial stage of the probe sliding on HOPG, the shear occurs between the silica asperities and graphite (Figure 5a). Because the silica is amorphous with some dangling bonds, such as —OH and Si—O—Si, the contact between the asperities and HOPG is impossible to form the incommensurate contact, which naturally leads to a high frictional force.

After the presliding, there are GNFs transferred onto the asperities, and as a result, the shear plane would be shifted from silica/graphite to graphene/graphite because of its lower shear strength (Figure 5b), which provides a favorable condition for the incommensurate contact. Obviously, the tribointeraction cannot control the rotation angle of these GNFs during the transfer process, and therefore, the transferred GNFs on each asperity should be oriented randomly as compared with the single crystalline HOPG substrate. As the lattices between the GNFs and HOPG are the same and perfectly rigid, it may form the overall incommensurate contact between the asperities and HOPG (Figure 5d). Thus, the condition of superlubricity is completely satisfied, which can lead to a significant reduction in the frictional force. In this case, the variation in the sliding angle cannot change the incommensurate contact, which is the reason why the superlubricity state is independent of the sliding orientation. Moreover, the superlubricity state was observed to be very stable (Figure 2c), which indicates that the transferred GNFs were firmly attached on the silica asperities after the presliding. The robustness may be related to the existence of interfacial adhesion between GNFs and silica, which is strong enough to prevent the GNFs from being detached from the probe during the sliding process (Figure S2, Supporting Information).

From Figure 5a,b, the adhesive force between the probe and HOPG originates from a series of asperities contact with HOPG, and therefore, the number (*N*_0_) of asperities contact with HOPG in the entire contact zone can be estimated by the following equation

(5) $$N_{0}=2F_{a}/3\pi rW_{\mathrm{AB}}$$

where *F*_a_ is the adhesive force and *r* is the average radius of the asperities (*r* ≈ 70 nm in Figure 1e). Thus the number of asperities in the contact zone is calculated as *N*_0_ ≈ 3. According to the Hertz contact theory, the calculated average contact pressure on these asperities in the superlubricity state can reach ≈700 MPa locally under an applied load of 460 nN. The shear strength between the transferred GNFs and graphite can be estimated by τ = *F*_s_/*A*, where *F*_s_ is the frictional force under an applied load and *A* is the real contact area in the contact zone, defined by *A* = *N*_0_ · *A_i_*, where *A_i_* is the contact area between each asperity and HOPG. According to the measured frictional force in Figure 1f, the shear strength between the transferred graphene nanoflake and graphite in their incommensurate contact is calculated as τ = 0.26−0.48 MPa. It is in the same level as the measured shear strength between the natural graphite layers (τ = 0.25−0.75 MPa) in ref. 19.

Because of the very weak vdW interaction between interlayers of layered materials, the superlubricity state can be easily achieved between their layers and sheets when they are in the incommensurate contact. The most impressive result in our study is the achievement of robust superlubricity between graphite and nonlayered materials via the formation of transferred GNFs by the tribointeractions. The friction coefficient of graphite can reduce to as low as 0.0003 by this method, which is much lower than that measured in other studies.[[qv: 3f]] This is because the frictional force is proportional to the real contact area, which is very small in our studies, owing to the asperity contact in the contact zone (not full contact). This result also exhibits that the superlubricity can be achieved on the rough surfaces, which is different from the superlubricity state achieved by the two atomically smooth surfaces, such as graphite or MoS_2_ sheets.[[qv: 3d,e]] Moreover, the superlubricity state achieved by this method was observed to be independent of the surface topography and roughness of silica, as shown in Figure 5e. Eight different silica particles glued onto the cantilever end to form the probes with random topography and roughness (Figure 5e, inset), and their frictional forces were measured as functions of normal loads. It is found that their frictional forces were all very small and their friction coefficients were always in the range of 0.0003–0.0013, reaching the superlubricity state. This result provides the possibility to achieve the superlubricity of graphite sliding against rough surface at the macroscale (on tribometer) by the tribointeractions.

## 3 Conclusion

In summary, our work has demonstrated that the superlubricity of graphite can be achieved by the formation of multiple transferred GNFs on the asperities of silica surfaces via the tribointeractions in the presliding process. The friction coefficient can reduce to as low as 0.0003 and remain very stable at a maximal local contact pressure of up to 700 MPa. The superlubricity state is independent of the sliding velocity, rotation angle, and surface roughness, which can be attributed to the extremely weak interaction and easy sliding between the transferred GNFs and graphite in their incommensurate contact. Our finding provides a possible method to achieve the robust superlubricity state of layered materials at the nanoscale by taking advantage of the tribointeractions that enables the transfer of 2D layered nanoflakes onto the opposite surface.

## 4 Experimental Section

*Frictional Force Microscopy Tests*: The friction measurements were performed using the Asylum Research MFP‐3D AFM in contact mode. A rectangular tipless cantilever (TL‐CONT) with a normal spring constant of 0.02–0.77 N m^−1^ and a lateral spring constant of 0.01–0.1 N m^−1^ was used, and the silica particles (*R* = 11.5 µm, elastic modulus ≈ 50 GPa) were glued onto the cantilever ends by using epoxy glue, yielding a SiO_2_ probe. Meanwhile, a HOPG (0.4° mosaic spread, elastic modulus ≈ 18 GPa) substrate was freshly cleaved by using the adhesive tape to give a clean atomically flat surface. The spring constant was determined by the frequency method,^20^ and the lateral detector sensitivity was obtained by using a diamagnetic lateral force calibrator.^21^ The frictional forces as functions of normal loads were measured by driving the probe sliding on HOPG as the load was increased (loading from 0 to 500 nN) under ambient conditions, at a temperature of 25 ± 2 °C and a relative humidity of 40–70%. The maximal diameter of contact area is about 130 nm at the applied load of 460 nN (estimated by the Hertz contact theory). Thus, the lateral scanning area was set in the range of 0.6 × 0.6 µm^2^ to 20 × 20 µm^2^ (it was equally divided into 16 parts as measured in the loading process), and the scanning velocity was set in the range of 0.3–30 µm s^−1^. The presliding process was performed at an applied load of 185 nN, and a scanning velocity of 10 µm s^−1^. The scanning area was set to 10 × 10 µm^2^ on different positions, and the presliding was stopped when the frictional force was observed to have a significant reduction. The distance for the presliding was varied from 10^2^ to 10^4^ µm, which was dependent on the SiO_2_ probe. The frictional forces before and after the presliding process were determined from the difference in the lateral force detector signal in one complete friction loop (20 friction loops were averaged for every load) and the lateral detector sensitivity.

*Surface Characterization*: The AFM image of the SiO_2_ probe was captured on the same AFM in tapping mode using a silicon tip array with a period of 3 µm. The topography of the SiO_2_ probe was measured on FESEM (HITACHI SU8220) under low voltage to protect the surface from damage. The TEM cross‐sectional sample was prepared by focused ion beam, picked out from the top of the probe. Cr film was first sputtered on the probe and Pt film was deposited subsequently as protective layers. The chemical composition on the top region of the probe (contact with HOPG) was measured by using XPS.

*Normal Force and Adhesive Force Measurement*: The normal force curves were obtained as the probe first approached the HOPG substrate and then retracted from the substrate with a velocity of 400 nm s^−1^. The adhesive forces were obtained by measuring the pull‐off forces as the probe detached from the HOPG substrate. The adhesive force mapping was obtained by measuring the pull‐off forces at 100 sites (1 µm^2^ per site) in an area of 10 × 10 µm^2^.

## Conflict of Interest

The authors declare no conflict of interest.

## Supporting information

Supplementary
