# Supplementary material for: Superlubricity of Graphite Induced by Multiple Transferred Graphene Nanoflakes
Source: Adv Sci (Weinh). 2018 Jan 3;5(3):1700616. doi: 10.1002/advs.201700616 (PMC5867060; doi:10.1002/advs.201700616)
Supplement: Supplementary file 1 — Supplementary [file ADVS-5-1700616-s001.pdf]

Copyright WILEY-VCH Verlag GmbH & Co. KGaA, 69469 Weinheim, Germany, 2018.

Supporting Information

for *Adv. Sci.,* DOI: 10.1002/advs.201700616

Superlubricity of Graphite Induced by Multiple Transferred Graphene Nanoflakes

Jinjin Li, Tianyang Gao, and Jianbin Luo*

Superlubricity of Graphite Induced by Multiple Transferred Graphene Nanoflakes

Jinjin Li, Tianyang Gao, Jianbin Luo*

State Key Laboratory of Tribology, Tsinghua University, Beijing, 100084, China

1. Frictional force properties in a large scanning area

It should be mentioned that there are always some atomic steps on the cleaved HOPG in a large area due to the different thickness of cleaved layer. When the frictional force was measured in a large scanning area (e.g.10 × 10 μm^2), there would appear friction peaks caused by the geometric effect as the probe slid across the atomic steps, but it did not have influence on the superlow friction when the probe slid on the atomically smooth area. In this work, we tried to avoid the appearance of atomic steps in the whole scanning area by choosing a relative smaller scanning area.

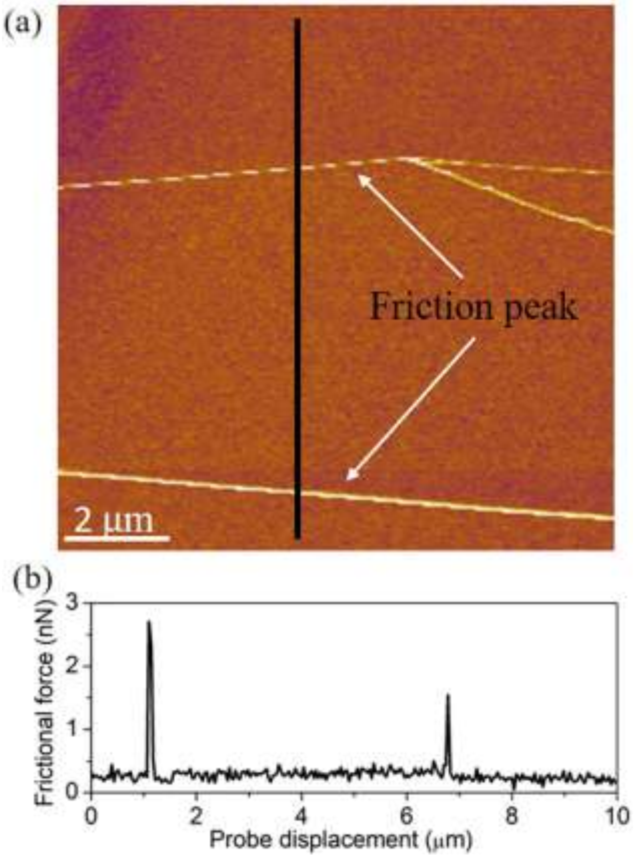

Figure S1 (a) Frictional force image in a large scanning area of 10 × 10 μm^2, showing the friction peaks caused by atomic steps and uniform frictional force in the atomically smooth area. The normal load is 200 nN and the sliding velocity is 20 μm/s (b) Frictional force profile along the black

line in (a), showing two friction peaks.

2. Explantion on the robustness of superlubricity

We designed an experiment here to explain the robustness; that is, the silica probe repeatedly approached HOPG and then detached from HOPG many times after the pre-sliding, and meanwhile the adhesive force was measured at each detachment. Figure S2 shows that the adhesive force always kept constant (close to the value in Figure 4b), even when the detachment exceeded 180 times. This result indicates that the GNFs were attached on the probe from the beginning to the end, otherwise the adhesive force would increase several times because of much higher adhesive force between silica and graphite (Figure 4a). Therefore it is inferred that the interfacial adhesion between GNFs and silica is strong enough to prevent the GNFs from being detached from the probe during the sliding process, which ensures the robustness of superlubricity.

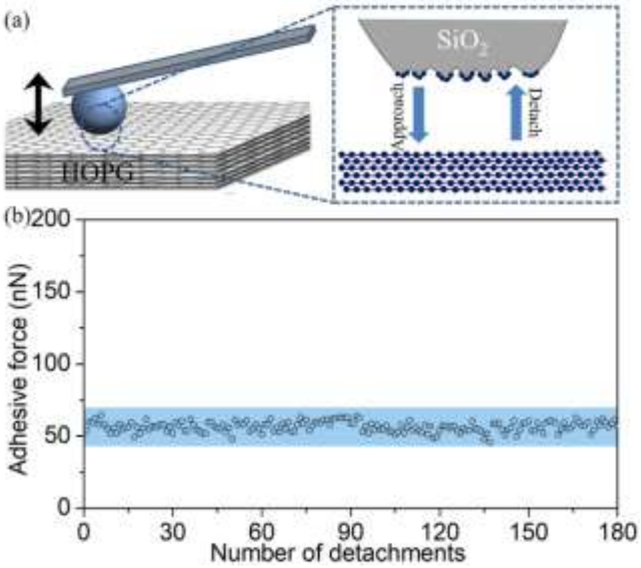

Figure S2 (a) Illustration of the desigined experiment. The probe repeatedly approached HOPG and then detached from HOPG many times after the pre-sliding. And the adhesive force between the probe and HOPG was measured at each detachment. (b) Adhesive forces measured under 180 times of detachments, showing no great increase with increasing number of detachments.
